# Comparative treatment persistence of upadacitinib vs. tofacitinib in psoriatic arthritis: a multicenter observational study from the BIRRA cohort

**DOI:** 10.3389/fmed.2026.1813319

**Published:** 2026-07-08

**Authors:** Alessandro Conforti, Martina Gentile, Valerio Cipolloni, Linda Lucchetti, Marta Priora, Andrea Becciolini, Eleonora Celletti, Miriam Di Penta, Alberto Lo Gullo, Marino Paroli, Elena Bravi, Romina Andracco, Valeria Nucera, Francesca Ometto, Federica Lumetti, Antonella Farina, Matteo Colina, Viviana Ravagnani, Palma Scolieri, Maddalena Larosa, Elisa Visalli, Olga Addimanda, Rosetta Vitetta, Alessandro Volpe, Alessandra Bezzi, Francesco Girelli, Aldo Biagio Molica Colella, Rosalba Caccavale, Eleonora Di Donato, Giuditta Adorni, Daniele Santilli, Gianluca Lucchini, Eugenio Arrigoni, Emanuela Sabatini, Ilaria Platè, Natalia Mansueto, Aurora Ianniello, Enrico Fusaro, Maria Chiara Ditto, Vincenzo Bruzzese, Dario Camellino, Gerolamo Bianchi, Francesca Serale, Rosario Foti, Giorgio Amato, Francesco De Lucia, Ylenia Dal Bosco, Roberta Foti, Massimo Reta, Alessia Fiorenza, Guido Rovera, Antonio Marchetta, Maria Cristina Focherini, Fabio Mascella, Simone Bernardi, Gilda Sandri, Dilia Giuggioli, Carlo Salvarani, Veronica Franchina, Francesco Molica Colella, Giulio Ferrero, Mirco Magnani, Marta Maset, Camilla Mazzanti, Gianluca Santoboni, Claudio Angrisani, Massimiliano De Simone, Bernd Raffeiner, Simone Parisi, Alarico Ariani

**Affiliations:** 1 Rheumatology Health Care, ASL Roma, Rome, Italy; 2 Ospedale San Paolo ASL ROMA 4, Pharmacology Unit, Civitavecchia, Italy; 3 Rheumatology Day Hospital and Outpatient Clinic, ASL CN1, Cuneo, Italy; 4 Internal Medicine and Rheumatology Unit, University Hospital of Parma, Parma, Italy; 5 Rheumatology Unit, Clinica Medica Institute, SS Annunziata Hospital, Chieti, Italy; 6 UOSD Reumatologia Ospedale Papardo, Messina, Italy; 7 Department of Clinical, Anesthesiological and Cardiovascular Sciences, Sapienza University of Rome, Italy; 8 Rheumatology Unit, Imperia Hospital, Imperia, Italy; 9 Rheumatology Outpatient Unit, ASL Novara, Novara, Italy; 10 Rheumatology Outpatient Clinic, Azienda ULSS 6 Euganea, Padova, Italy; 11 Rheumatology Unit, Azienda USL of Modena and AOU Policlinico of Modena, Modena, Italy; 12 Internal Medicine Unit, Rheumatology Outpatient Clinic, Augusto Murri Hospital, Fermo, Italy; 13 Servizio di Reumatologia, UOC Medicina Interna A, Dipartimento Medico-Oncologico, Ospedale Santa Maria della Scaletta, Imola, Bologna, Italy; 14 Alma Mater Studiorum, Dipartimento di Scienze Biomediche e Neuromotorie (DIBIDEM), Università di Bologna, Bologna, Italy; 15 Rheumatology Unit, Santa Chiara Hospital, APSS—Trento, Trento, Italy; 16 Rheumatology Unit, Department of Medical Specialties, Nuovo Regina Margherita Hospital, Rome, Italy; 17 Division of Rheumatology, Department of Medical Specialties, La Colletta Hospital, ASL 3 Genova, Genoa, Italy; 18 Rheumatology Unit, Policlinico San Marco Hospital, Catania, Italy; 19 Rheumatology Unit, AUSL of Bologna—Policlinico Sant'Orsola—AOU—IRCCS of Bologna, Bologna, Italy; 20 Rheumatology Unit, ASL VC Sant'Andrea Hospital, Vercelli, Italy; 21 Rheumatology Unit, IRCCS Sacro Cuore Don Calabria Hospital, Negrar di Valpolicella, Italy; 22 Internal Medicine and Rheumatology Unit, ASL Romagna—Rimini, Rimini, Italy; 23 Rheumatology Unit, G.B. Morgagni—L. Pierantoni Hospital, Forlì, Italy; 24 Rheumatology Unit, Division of Internal Medicine, A.O. Papardo, Messina, Italy; 25 Rheumatology Department, Azienda Ospedaliero-Universitaria Città della Salute e della Scienza di Torino, Turin, Italy; 26 Rheumatology Unit, University of Modena and Reggio Emilia, Modena, Italy; 27 Medical Oncology Unit, A.O. Papardo, Messina, Italy; 28 Internal Medicine Unit, Milano-Bicocca University, Milan, Italy; 29 Unit of Diagnostic and Interventional Radiology, Santa Corona Hospital, Pietra Ligure, Italy; 30 UOSD Reumatologia, Dipartimento Area Medica, Ospedale di Belluno, AULSS 1 Dolomiti, Belluno, Italy; 31 Center for the Diagnosis and Therapy of Autoimmune Rheumatological Diseases, Viterbo, Italy; 32 UOC Reumatologia, Department of Rheumatology, Bolzano, Italy

**Keywords:** effectiveness, observational studies, psoriasic arthritis, tofacinib, upadacitin

## Abstract

**Background:**

Real-world comparative data on treatment persistence of upadacitinib and tofacitinib in psoriatic arthritis remain limited. Because persistence reflects treatment durability, tolerability, and perceived benefit in routine practice, it may serve as a pragmatic measure of real-world effectiveness.

**Objective:**

This study aimed to compare real-world treatment persistence between upadacitinib and tofacitinib in patients with psoriatic arthritis and to identify predictors of treatment discontinuation using data from the Italian multicenter BIRRA cohort.

**Methods:**

In this retrospective, multicenter, observational study, PsA patients treated with UPA and/or TOFA were enrolled from 34 rheumatology centers. Baseline demographics, treatment details, and disease activity (DAPSA) were collected. Treatment persistence was evaluated using Kaplan-Meier survival analysis. Cox proportional hazards models identified predictors of discontinuation, including sex, age, treatment line, prescription year, concomitant csDMARDs/steroids, PsA subtype (peripheral, axial, or mixed), and prior or current JAK inhibitor (JAKi) use.

**Results:**

Among 181 enrolled patients (UPA *n* = 124; TOFA *n* = 57), retention rates at 6, 12, and 18 months were 86%, 68%, and 54% for UPA and 78%, 60%, and 60% for TOFA (*p* = 0.7). Concomitant csDMARD therapy (HR: 1.92; 95% CI: 1.04–3.54; *p* = 0.037) and later-line treatment (HR: 1.17; 95% CI: 1.01–1.35; *p* = 0.034) were independently associated with higher discontinuation risk. No statistically significant differences were observed between the two JAK inhibitors.

**Conclusion:**

UPA demonstrated a slightly longer persistence than TOFA, though the difference was not statistically significant after adjustment. Concomitant csDMARDs and later treatment lines significantly reduced persistence. These results suggest that PsA treatment retention may be influenced more by patient- and treatment-related factors than by the specific JAK inhibitor prescribed.

## Highlights

- A real-world multicenter study found no significant difference in drug persistence between upadacitinib and tofacitinib after adjustment for clinical factors.
- The use of csDMARDs in combination and later-line therapy were independent predictors of treatment discontinuation.

## Introduction

Psoriatic arthritis (PsA) is a chronic inflammatory disease associated with psoriasis, affecting approximately 1% of the global population (1). About 30% of individuals with psoriasis develop PsA, which often impairs function and reduces quality of life (2). The disease affects peripheral joints, the axial skeleton, entheses, and the skin or nails (3). Its development is driven by genetic, immune, and environmental factors (4). Treatment options have advanced with the introduction of targeted synthetic disease-modifying antirheumatic drugs (tsDMARDs), including Janus kinase (JAK) inhibitors (5). Tofacitinib, a pan-JAK inhibitor, and upadacitinib, a JAK1-selective agent, both showed efficacy in randomized controlled trials (2, 5). However, real-world comparative studies in PsA remain few (6). Meta-analyses have produced inconsistent results due to the heterogeneity in defining persistence and to limited follow-up (7). A U.S. study in rheumatoid arthritis reported higher persistence with upadacitinib (8) but these findings may not apply to PsA due to the differences between these chronic arthritis. As far as we know there are not studies contrasting upadacitinib and tofacitinib in PsA.

This real-world study aimed to compare treatment persistence between tofacitinib and upadacitinib in patients with PsA and to identify clinical and treatment-related predictors of discontinuation. In this context, persistence was considered a pragmatic measure of real-world effectiveness, reflecting the combined influence of efficacy, tolerability, patient preference, and clinician decision-making in routine care.

## Materials and methods

This retrospective, multicenter, observational study was conducted within the framework of the BIRRA (BIologics Retention Rate Assessment) cohort. The BIRRA initiative is designed to evaluate real-world persistence and predictors of treatment discontinuation among patients with inflammatory arthritis in Italy.

The study protocol was reviewed and approved by the Comitato Etico dell'Area Vasta Emilia Nord (Protocol Code: 34713; Approval Date: March 5, 2021), which served as the lead coordinating ethics committee. Each participating rheumatology center obtained additional local approval in compliance with national regulations. All procedures adhered to the principles of the Declaration of Helsinki (2013 revision). Written informed consent was obtained from all participants prior to inclusion.

Eligible participants included consecutive adult PsA patients recruited from 34 specialized rheumatology centers across Italy. The inclusion criteria comprised: (a) confirmed PsA diagnosis based on the CASPAR classification, (b) initiation of upadacitinib or tofacitinib therapy on or after January 1, 2018, (c) documented records of treatment commencement and discontinuation dates. Exclusion criteria involved: (a) Patients treated with JAK inhibitors for non-articular indications (e.g., psoriasis, inflammatory bowel disease, uveitis), (b) patients missing baseline data on treatment start or discontinuation dates, (c) concomitant participation in interventional clinical trials during follow-up.

The study population was divided into two cohorts based on the prescribed targeted synthetic DMARD (tsDMARD): upadacitinib (UPA) or tofacitinib (TOFA). Collected variables encompassed: demographic and clinical profiles (age, sex, disease duration at baseline, and PsA subtype—peripheral, axial, or mixed), therapeutic parameters [duration of tsDMARD exposure, concurrent use of conventional synthetic DMARDs (csDMARDs) and/or corticosteroids] and peripheral baseline disease activity [evaluated via the Disease Activity Index for Psoriatic Arthritis (DAPSA)].

Patients who received both tsDMARDs during follow-up (*n* = 15) were analyzed using a time-segmented approach. Each treatment period was assigned to the corresponding drug, and switching from one JAK inhibitor to the other was treated as a discontinuation event for the first drug and as a new treatment episode for the subsequent drug. The direction of switching, timing when available, and reason for switching were recorded descriptively.

Treatment discontinuation was defined as permanent cessation, switching to another biologic or JAK inhibitor, or a treatment gap of more than 60 consecutive days without reinitiation. Temporary interruptions due to surgery, infection, or administrative delays were not considered discontinuations. Patients lost to follow-up or remaining on treatment at the last available visit were censored at that point. Reasons for discontinuation were categorized as follows. “Lack of efficacy” was defined as primary non-response or insufficient clinical improvement leading to treatment cessation during the initial treatment period. “Loss of efficacy” was defined as secondary loss of clinical benefit after an initial response or period of disease control. Adverse events were defined as treatment discontinuations due to tolerability or safety concerns, excluding major adverse cardiovascular events and malignancy when reported separately.

Patients who were lost to follow-up or discontinued treatment due to skin-related adverse events were right-censored in the analysis. Descriptive statistics for continuous variables were expressed as median and interquartile range (IQR), whereas categorical data were presented as proportions. Treatment persistence, a proxy for effectiveness, was compared between groups using Kaplan-Meier survival analysis and log-rank testing.

To identify predictors of drug retention, a Cox proportional hazards model was employed, incorporating covariates such as age, sex, tsDMARD type, PsA subtype, disease duration, DAPSA score, treatment initiation year, and concomitant csDMARD or corticosteroid use. Intergroup differences were assessed using the Mann-Whitney U test (continuous variables) or Pearson's chi-square test (categorical variables), as appropriate. Statistical significance was defined as a two-tailed ^*^p^*^-value <0.05. All computations were executed in Jamovi (version 2.6; https://www.jamovi.org). Missing data were assessed by variable and treatment group. No imputation was performed because missingness was limited to baseline DAPSA. Analyses involving DAPSA were conducted using complete available cases, whereas other baseline and treatment variables were analyzed in the full cohort.

Hazard ratios (HR) and corresponding 95% confidence intervals (95% CI) were reported for each predictor. Because baseline sex distribution differed substantially between the treatment groups, an additional propensity score-adjusted sensitivity analysis was performed to assess the robustness of the primary comparison. The propensity score included sex, age, treatment line, year of prescription, concomitant csDMARD use, concomitant corticosteroid therapy, PsA subtype, and JAK inhibitor-naïve status. The association between treatment group and discontinuation was then re-estimated after adjustment for the propensity score.

This study was conducted and reported in adherence to the STROBE (Strengthening the Reporting of Observational Studies in Epidemiology) guidelines. A completed STROBE checklist is available as Supplementary material.

## Results

### Patient characteristics

A total of 181 patients with psoriatic arthritis treated with at least one targeted synthetic disease-modifying antirheumatic drug were included from the participating BIRRA centers. Overall, 124 treatment episodes were assigned to upadacitinib and 57 to tofacitinib. Fifteen patients received both Janus kinase inhibitors during follow-up; 12 switched from tofacitinib to upadacitinib, whereas 3 switched from upadacitinib to tofacitinib.

Baseline characteristics are summarized in Table 1. The two treatment groups showed a significant imbalance in sex distribution, with a higher proportion of males in the upadacitinib group and a higher proportion of females in the tofacitinib group. Concomitant corticosteroid use was also more frequent in the upadacitinib group. Age, PsA duration, PsA subtype, baseline DAPSA, concomitant csDMARD use, and follow-up duration were broadly comparable between groups. Baseline DAPSA was the only variable with missing data and was unavailable in 36 patients, including 24 patients in the upadacitinib group and 12 patients in the tofacitinib group. No imputation was performed.

**Table 1:** Baseline characteristics of PsA patients.

Variable	UPA (*n* = 124)	TOFA (*n* = 57)	*p*-value
**Male sex**, ***n*** **(%)**	88 (71.0)	12 (21.1)	<0.001^*^
**Female sex**, ***n*** **(%)**	36 (29.0)	45 (78.9)	
**Age, median (IQR), years**	58 (49–63)	56 (47–61)	0.42
**PsA duration, median (IQR), months**	63 (36–123)	63 (32–144)	0.87
**Treatment line, median (IQR)**	4 (3–5)	4 (3–5)	
**PsA subtype**, ***n*** **(%)**			0.46
•Peripheral	69 (55)	35 (61)	
•Axial	12 (10)	5 (9)	
•Mixed	43 (35)	17 (30)	
**Baseline DAPSA, median (IQR)**	25.8 (21.0–33.0)	25.0 (21.0–28.7)	0.58
**Concomitant csDMARD use**, ***n*** **(%)**	43 (35)	17 (30)	0.49
**Concomitant corticosteroid use**, ***n*** **(%)**	48 (39)	12 (21)	0.019
**Follow-up, median (IQR), days**	190 (78–382)	185 (81–374)	0.73

Data missing in 26 (^*^) patients. Baseline DAPSA data were missing in 36 patients: 24 in the UPA group and 12 in the TOFA group. No imputation was performed. Analyses including DAPSA were conducted using available cases. *p*-values were obtained using the Mann–Whitney *U*-test for continuous variables and Pearson's χ^2^ test for categorical variables. IQR, interquartile range; DAPSA, Disease Activity in Psoriatic Arthritis score; csDMARD, conventional synthetic disease-modifying antirheumatic drug; PsA, psoriatic arthritis. N/A, not applicable; ns, not significant; *p* < 0.05 considered statistically significant. Data missing for DAPSA in 26 patients (indicated by asterisk in the main text).

### Treatment persistence

The median observation period was 190 days, with an interquartile range of 78–382 days, and the total follow-up duration was 44,295 patient-days. Kaplan–Meier analysis showed no statistically significant difference in treatment persistence between upadacitinib and tofacitinib.

**Figure 1:**
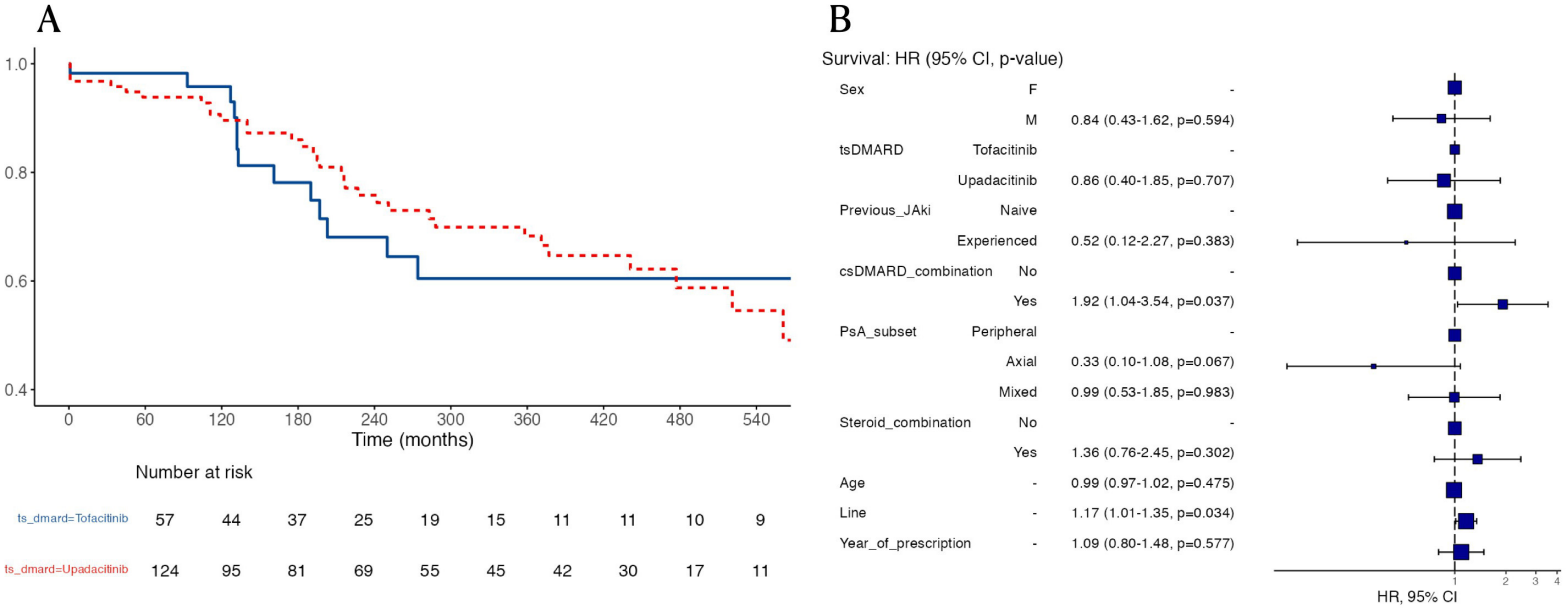
Treatment persistence of upadacitinib and tofacitinib in patients with psoriatic arthritis. **(A)** Kaplan–Meier curves comparing drug retention between upadacitinib and tofacitinib, censored at 540 days because of the limited number of patients remaining at risk beyond this time point. **(B)** Cox proportional hazards regression plot showing predictors of treatment discontinuation. UPA, upadacitinib; TOFA, tofacitinib; csDMARD, conventional synthetic disease-modifying antirheumatic drug; HR, hazard ratio; CI, confidence interval.

Retention rates in the upadacitinib group were 86%, 68%, and 54% at 180, 360, and 540 days, respectively. In the tofacitinib group, retention rates were 78%, 60%, and 60% at the same time points. The difference between groups was not statistically significant by log-rank testing (*p* = 0.7) (Figure 1A). Median drug survival was not reached in either group during the available follow-up period because more than half of patients remained on treatment within the observation window. Therefore, persistence was summarized using retention probabilities at 180, 360, and 540 days.

Because the number of patients remaining at risk at 540 days was small, particularly in the tofacitinib group, estimates at this later time point should be interpreted cautiously.

### Predictors of treatment discontinuation

In the multivariable Cox regression analysis, disease duration was excluded because of significant correlation with age and treatment line. Among the remaining covariates, concomitant csDMARD therapy and later treatment line were independently associated with a higher risk of treatment discontinuation.

Concomitant csDMARD therapy was associated with an increased risk of discontinuation compared with no csDMARD combination therapy (HR 1.92; 95% CI 1.04–3.54; *p* = 0.037). Later treatment line was also associated with increased discontinuation risk (HR 1.17; 95% CI 1.01–1.35; *p* = 0.034). No significant association with discontinuation was observed for sex, treatment type, previous JAK inhibitor exposure, PsA subset, corticosteroid use, age, or year of prescription (Figure 1B).

### Sensitivity analysis

Because of the marked baseline imbalance in sex distribution between treatment groups, a propensity score-adjusted sensitivity analysis was performed. The propensity score included sex, age, treatment line, year of prescription, concomitant csDMARD use, concomitant corticosteroid therapy, PsA subtype, and JAK inhibitor-naïve status.

After adjustment using the propensity score, upadacitinib was not associated with a significantly different risk of discontinuation compared with tofacitinib (HR 0.97; 95% CI 0.50–1.91; *p* = 0.935). This result confirmed the robustness of the primary analysis and supported the absence of a significant difference in treatment persistence between the two JAK inhibitors.

### Reasons for discontinuation

Reasons for treatment discontinuation are summarized in Table S2. In the upadacitinib group, 35 patients discontinued treatment. The most common reasons were lack of efficacy in 13 patients (37.1%), loss of efficacy in 11 patients (31.4%), adverse events excluding major adverse cardiovascular events and malignancy in 7 patients (20.0%), and infections in 4 patients (11.4%).

In the tofacitinib group, 17 patients discontinued treatment. Discontinuation was attributed to lack of efficacy in 11 patients (64.7%), loss of efficacy in 5 patients (29.4%), and adverse events excluding major adverse cardiovascular events and malignancy in 1 patient (5.9%). No infections were reported as a reason for discontinuation in the tofacitinib group. No deaths occurred during follow-up. There were no statistically significant differences in the distribution of discontinuation reasons between the two treatment groups.

### Treatment switching

Fifteen patients switched between the two JAK inhibitors during follow-up. Most switches occurred from tofacitinib to upadacitinib, observed in 12 patients, whereas 3 patients switched from upadacitinib to tofacitinib. These patients were analyzed by treatment episode. Switching was considered a discontinuation event for the first JAK inhibitor and the beginning of a new treatment episode for the subsequent JAK inhibitor.

When available, the main reasons for switching were lack of efficacy, loss of efficacy, or intolerance/adverse events. Given the small number of switching patients, switching patterns were described descriptively and were not used for separate comparative statistical modeling.

## Discussion

This multicenter real-world study compared treatment persistence between UPA and TOFA in patients with PsA. Persistence was used as a pragmatic marker of real-world effectiveness, reflecting treatment durability, tolerability, patient preference, and clinician decision-making. Overall, no statistically significant difference in persistence was observed between UPA and TOFA, and this finding remained consistent after multivariable and propensity score-adjusted analyses. Instead, treatment discontinuation was more strongly associated with concomitant csDMARD use and later treatment line.

While UPA showed numerically better retention, this lost significance after multivariable adjustment is consistent with other real-world data. A Swedish retrospective study similarly found no substantial differences in biologic persistence, underlining the importance of patient heterogeneity and treatment exposure in shaping outcomes (9). Additionally, a network meta-analysis showed no difference in discontinuation risk between UPA and TOFA, linking retention more with disease subtype and prior therapies (10).

These findings are consistent with recent real-world and multicenter studies evaluating JAK inhibitors and upadacitinib in PsA, which suggest that persistence is influenced by prior biologic exposure, disease phenotype, comorbidity burden, and treatment sequencing rather than by drug choice alone (15, 16).

JAK1 inhibition likely plays a critical role in PsA pathogenesis. Both UPA and TOFA partially inhibit JAK1, possibly explaining their similar effectiveness. JAK1 mediates signaling of key PsA cytokines such as IL-6, IL-23, and interferons, affecting both joint and skin inflammation (11–13). Despite differences in their selectivity profiles, the shared ability to inhibit JAK1 may underlie their comparable clinical outcomes.

Real-world effectiveness of UPA has shown favorable retention and minimal disease activity rates (14). Italian multicenter data reported 12-month UPA survival with efficacy across axial and peripheral domains (15). Discontinuation predictors—treatment line and csDMARD co therapy align with prior studies. Longer disease duration, often linked with later treatment lines, tends to reduce therapeutic response due to accrued joint damage and pharmacologic tolerance (8). Higher dropout was seen in patients with multiple prior biologics even after adjusting for disease activity (16). The 540-day retention estimates should be interpreted with caution because the number of patients remaining at risk was small at this time point. Therefore, the later persistence estimates are best viewed as exploratory rather than definitive.

Interestingly, csDMARD co-therapy correlated with lower drug survival, challenging conventional views. Though conventional DMARDs such as methotrexate is often used with biologics in RA, its utility in PsA, especially for axial or entheseal disease, is less clear (17). A study by Skouvaklidou et al. linked csDMARD use with reduced UPA persistence, possibly reflecting more aggressive disease in these patients (18).

The association between concomitant csDMARD therapy and lower drug persistence observed here deserves further consideration. Although csDMARDs such as methotrexate are often combined with biologics in rheumatoid arthritis to improve immunogenicity and durability, their additive benefit in PsA, particularly for axial or entheseal involvement, remains uncertain. Our data suggest that csDMARD co-therapy may reflect a subgroup with more aggressive, treatment-resistant disease rather than a true pharmacologic interaction leading to poorer retention.

No significant effect was found for baseline DAPSA, age, sex, or prior JAKi use. This suggests treatment context, particularly prior therapy and polypharmacy, outweighs baseline biology in determining persistence. This supports calls for personalized PsA treatment strategies based on disease phenotype and history (19).

Despite regulatory concerns, no cardiovascular events occurred in this cohort. The EMA and FDA have warned of cardiovascular and malignancy risks with JAKi, particularly in older or multimorbid patients. However, a meta-analysis found no consistent cardiovascular risk increase in PsA JAKi use, suggesting patient selection and baseline risk are more relevant than the drugs themselves (3). Our findings support the real-world safety of JAK inhibitors when used judiciously.

From a clinical perspective, persistence alone may be inadequate for JAKi comparison. Disease subtype, comorbidities, and prior treatments offer better clinical guidance. Nonetheless, the comparable persistence of UPA and TOFA in our real-world setting provides important reassurance to clinicians when sequencing targeted therapies in PsA. In particular, the modest trend toward higher retention with UPA may warrant further investigation in prospective registries.

The high dropout rates especially with later-line TOFA may reflect reduced JAKi responsiveness post-biologic failure. Biomarker-guided treatment may improve this, though PsA biomarkers remain underdeveloped.

These results affirm the value of real-world data alongside RCTs. Trials like SELECT-PsA2 validate UPA in refractory cases (20), but real-world settings capture broader patient variability. Our data collection spans different therapy lines and practice settings across Italy, enhancing external validity.

Strengths include multicenter design, detailed confounder adjustment, and clear documentation of discontinuation reasons—improving interpretation and shared decision-making.

Limitations include the retrospective design, potential residual confounding, and relatively short follow-up period. The substantial imbalance in sex distribution between the UPA and TOFA groups may have introduced residual confounding, although sex was not independently associated with discontinuation in the multivariable Cox model and a propensity score-adjusted sensitivity analysis confirmed the absence of a significant difference between treatments. The limited number of TOFA-treated patients and the small number of patients remaining at risk at 540 days may have reduced the precision and stability of later persistence estimates. In addition, treatment switching between TOFA and UPA may have influenced drug survival estimates, particularly because switching may reflect inefficacy, intolerance, treatment availability, or clinician preference. Finally, missing baseline DAPSA data in 36 patients limited complete-case adjustment for disease activity.

Our study adds to the growing real-world literature demonstrating that treatment persistence in PsA reflects both drug characteristics and patient context, supporting a pragmatic, individualized approach to JAK inhibitor selection and sequencing.

## Conclusion

This first real-world head-to-head comparison of JAK inhibitors in PsA found no significant difference in treatment persistence between UPA and TOFA. Retention was more strongly associated with co-therapy and treatment history than with the specific agent. In this multicenter real-world cohort of patients with PsA, treatment persistence did not differ significantly between UPA and TOFA. Discontinuation was more strongly associated with concomitant csDMARD use and later treatment line than with the specific JAK inhibitor prescribed. These findings suggest broadly comparable real-world persistence of UPA and TOFA, although the retrospective design, baseline imbalance in sex distribution, treatment switching, and limited numbers at later follow-up require cautious interpretation. Further prospective registry studies are needed to confirm these observations and better define patient-level predictors of JAK inhibitor persistence in PsA.

## Data Availability

The original contributions presented in the study are included in the article/supplementary material, further inquiries can be directed to the corresponding author.
