# Supplementary material for: Comparative treatment persistence of upadacitinib vs. tofacitinib in psoriatic arthritis: a multicenter observational study from the BIRRA cohort
Source: Front Med (Lausanne). 2026 Jul 8;13:1813319. doi: 10.3389/fmed.2026.1813319 (PMC13388746; doi:10.3389/fmed.2026.1813319)
Supplement: Supplementary file 1 [file Data_Sheet_1.docx]

**Supplementary Material**

**Table S1: STROBE Statement: Checklist of Items That Should Be Included in Reports of Cohort Studies**

| **Item No.** | **Recommendation** | **Section in Manuscript** |
| --- | --- | --- |
| **Title and Abstract** |  |  |
| 1(a) | Indicate the study’s design with a commonly used term in the title or the abstract. | Title page, Abstract (“multicenter observational study”) |
| 1(b) | Provide an informative and balanced summary of what was done and what was found. | Abstract (structured format) |
| **Introduction** |  |  |
| 2 | Explain the scientific background and rationale for the investigation being reported. | Introduction, ¶1–2 |
| 3 | State specific objectives, including any prespecified hypotheses. | Introduction, final paragraph |
| **Methods** |  |  |
| 4 | Present key elements of study design early in the paper. | Materials and Methods, first paragraph |
| 5 | Describe the setting, locations, and relevant dates, including periods of recruitment, exposure, follow-up, and data collection. | Materials and Methods, “Study design and participants” |
| 6(a) | Give the eligibility criteria, and the sources and methods of selection of participants. Describe methods of follow-up. | Materials and Methods, inclusion/exclusion criteria |
| 6(b) | For matched studies, give matching criteria and number of exposed and unexposed. | N/A (not applicable, no matching) |
| 7 | Clearly define all outcomes, exposures, predictors, potential confounders, and effect modifiers. Give diagnostic criteria if applicable. | Materials and Methods, “Variables and data collection” |
| 8 | For each variable of interest, give sources of data and details of methods of assessment (measurement). Describe comparability of assessment methods if more than one group. | Materials and Methods, “Variables and data collection” |
| 9 | Describe any efforts to address potential sources of bias. | Materials and Methods (“Exclusion criteria” and “Statistical analysis”) |
| 10 | Explain how the study size was arrived at. | Described implicitly as inclusion of all eligible cases in registry |
| 11 | Explain how quantitative variables were handled in the analyses. If applicable, describe which groupings were chosen and why. | Statistical analysis section |
| 12(a) | Describe all statistical methods, including those used to control for confounding. | Statistical analysis section |
| 12(b) | Describe any methods used to examine subgroups and interactions. | Not applicable (no subgroup modeling) |
| 12(c) | Explain how missing data were addressed. | Missing data were limited to baseline DAPSA; no imputation was performed, and available-case analysis was used for models including DAPSA. |
| 12(d) | If applicable, explain how loss to follow-up was addressed. | Patients lost to follow-up censored |
| 12(e) | Describe any sensitivity analyses. | Not applicable (none performed) |
| **Results** |  |  |
| 13(a) | Report numbers of individuals at each stage of study—e.g., numbers potentially eligible, examined for eligibility, confirmed eligible, included in the study, completing follow-up, and analyzed. | Results, first paragraph |
| 13(b) | Give reasons for non-participation at each stage. | Described in exclusion criteria and flow narrative |
| 13(c) | Consider use of a flow diagram. | Fig. 1 (Kaplan-Meier and model) |
| 14(a) | Give characteristics of study participants (e.g., demographic, clinical, social) and information on exposures and potential confounders. | Results, first paragraph, Table 1 |
| 14(b) | Indicate number of participants with missing data for each variable of interest. | Table 1 footnote (IQR missing data note) |
| 14(c) | Summarize follow-up time (e.g., average and total amount). | Results, first paragraph (median and total follow-up duration) |
| 15 | Report numbers of outcome events or summary measures over time. | Results, treatment persistence paragraph |
| 16(a) | Give unadjusted estimates and, if applicable, confounder-adjusted estimates and their precision (e.g., 95% CI). Make clear which confounders were adjusted for and why they were included. | Results, multivariate Cox regression |
| 16(b) | Report category boundaries when continuous variables were categorized. | Not applicable (continuous variables kept as continuous) |
| 16(c) | If relevant, consider translating estimates of relative risk into absolute risk for a meaningful time period. | Not applicable |
| 17 | Report other analyses done—e.g., analyses of subgroups and interactions, and sensitivity analyses. | Not applicable |
| **Discussion** |  |  |
| 18 | Summarize key results with reference to study objectives. | Discussion, opening paragraph |
| 19 | Discuss limitations of the study, taking into account sources of potential bias or imprecision. Discuss direction and magnitude of any potential bias. | Discussion, last paragraphs |
| 20 | Give a cautious overall interpretation of results considering objectives, limitations, multiplicity of analyses, results from similar studies, and other relevant evidence. | Discussion, throughout; esp. link to RHEI studies |
| 21 | Discuss the generalizability (external validity) of the study results. | Discussion, last paragraph |
| **Other Information** |  |  |
| 22 | Give the source of funding and the role of the funders for the present study. | Title page, “Funding” |
| 23 | Provide statement of conflicts of interest, acknowledgments, and author contributions. | End sections: “Conflicts of Interest,” “Acknowledgments,” “Author Contributions” |
| 24 | Mention ethical approval and informed consent. | Materials and Methods, “Ethics approval” |
| 25 | Include data availability and open data sharing statement. | “Open Data and Open Access Statement” |

**Table S2: Reasons for treatment discontinuation by drug**

| Reason for discontinuation | Upadacitinib (n = 124) | Tofacitinib (n = 57) | p-value |
| --- | --- | --- | --- |
| Lack of efficacy | 13 (37.1%) | 11 (64.7%) | 0.09 |
| Loss of efficacy | 11 (31.4%) | 5 (29.4%) | 0.88 |
| Adverse events *(excluding MACE and malignancy)* | 7 (20.0%) | 1 (5.9%) | 0.18 |
| Infections | 4 (11.4%) | 0 (0%) | 0.12 |
| Total discontinuations | 35 (28.2% of UPA group) | 17 (29.8% of TOFA group) | 0.84 |
